# Antiviral Activity of* Fridericia formosa* (Bureau) L. G. Lohmann (Bignoniaceae) Extracts and Constituents

**DOI:** 10.1155/2017/6106959

**Published:** 2017-05-29

**Authors:** Geraldo Célio Brandão, Erna G. Kroon, José D. Souza Filho, Alaíde Braga Oliveira

**Affiliations:** ^1^Departamento de Farmácia, Escola de Farmácia, Universidade Federal de Ouro Preto, Campus Morro do Cruzeiro, 35.400-000 Ouro Preto, MG, Brazil; ^2^Departamento de Microbiologia, ICB, Universidade Federal de Minas Gerais, Av. Antônio Carlos 6627, 31.270-901 Belo Horizonte, MG, Brazil; ^3^Departamento de Química, ICEX, Universidade Federal de Minas Gerais, Av. Antônio Carlos 6627, 31.270-901 Belo Horizonte, MG, Brazil; ^4^Departamento de Produtos Farmacêuticos, Faculdade de Farmácia, Universidade Federal de Minas Gerais, Av. Antônio Carlos 6627, 31.270-901 Belo Horizonte, MG, Brazil

## Abstract

A phytochemical study of* Fridericia formosa *(Bignoniaceae) ethanol extracts of leaves, stems, and fruits was guided by in vitro assays against vaccinia virus Western Reserve (VACV-WR), human herpes virus 1 (HSV-1), murine encephalomyocarditis virus (EMCV), and dengue virus type 2 (DENV-2) by the MTT method. All the ethanol extracts were active against DENV-2, HSV-1, and VACV-WR with best results for the fruits extract against DENV-2 (SI > 38.2). For VACV-WR and HSV-1, EC50 values > 200 *μ*g mL^−1^ were determined, while no inhibition of the cytopathic effect was observed with EMCV. Five compounds were isolated and identified as the C-glucosylxanthones mangiferin (**1**), 2′-*O-trans*-caffeoylmangiferin (**2**), 2′-*O-trans*-coumaroylmangiferin (**3**), 2′-*O*-trans-cinnamoylmangiferin (**5**), and the flavonoid chrysin (**4**). The most active compound was 2′-*O-trans*-coumaroylmangiferin (**3**) with SI > 121.9 against DENV-2 and 108.7 for HSV-1. These results indicate that mangiferin cinnamoyl esters might be potential antiviral drugs.

## 1. Introduction

Viral infections represent a current problem accounting for severe damage to human health and economic losses in livestock [1]. Some viral diseases such as dengue or dengue fever (DF), herpes, smallpox, and encephalomyocarditis have a high impact in public health in the tropical and subtropical regions of the world [1].

Dengue virus belonging to the Flaviviridae family,* Flavivirus* genus, is responsible for Dengue fever (DF) and is considered the most common arboviral disease of humans. It is estimated that 390 million cases occur every year around the world and it is endemic in more than 100 countries, including the Americas, Southeast Asia, and Western Pacific, regions most seriously affected [1, 2]. No effective drug as well as no vaccine is available for human use. The need for a safe and efficient approach either for treatment or prevention of DF has been considered a global priority [1, 3].

HSV belongs to the family Herpesviridae and the subfamily Alphaherpesvirinae and is characterized by neurovirulence, latency, and reactivation. The prevalence of HSV infection has increased in recent years, making it a highly relevant public health issue. Early detection and treatment are of paramount importance for disease control [4].

Encephalomyocarditis virus (EMCV) family Picornaviridae, genus* Cardiovirus*, is a group of closely related virus species with a wide host range. Infections with EMCV are associated with sporadic cases and outbreaks of myocarditis and encephalitis in domestic pigs, in nonhuman primates and other mammalian species. There are few reports of cases of human infection by EMCV [5].

Vaccinia (VACV) is a virus of the genus* Orthopoxvirus* of the family Poxviridae that, in humans, causes nonlethal, pustular, and localized disease. The vaccinia virus does not have natural hosts, but cases of bovine and human infection by vaccinia virus are reported in Brazil and India, causing economic losses and affecting health services [6, 7].

As part of a bioprospecting project, whose main goal is to discover potential antiviral natural products of plants from Brazilian Cerrado and Atlantic Forest biomes, we have screened several species of plants collected in the state of Minas Gerais [1, 8–12]. Among these,* Fridericia formosa* (Bureau) Sandwith was chosen for bioguided phytochemical investigation due to the good antiviral activity presented by the ethanol extracts of leaves, stems, and fruits.

## 2. Materials and Methods

### 2.1. Collection, Taxonomical Determination, and Processing of Plant Materials

*F. formosa* was collected in the municipality of Belo Horizonte, Minas Gerais, Brazil. The plant was taxonomically identified by Dr. J. A. Lombardi, Departamento de Botânica, Instituto de Biociências, UNESP, Rio Claro, Brazil. A voucher specimen was deposited at the BHCB/UFMG, Belo Horizonte, Minas Gerais, Brazil, under the number 23885.

### 2.2. Preparation of Extracts

After drying in air circulating oven at 40°C for 72 h, the plant material—136.3 g of leaves, 461.5 g of stems, and 13.8 g fruits—were ground and extracted by percolation with 96% EtOH at room temperature. The solvent was removed in a rotary evaporator under reduced pressure at 50°C, leaving dark residues—EEFFL, 34.2 g, EEFFS, 60.0 g, and EEFFF, 2.5 g—for leaves, stems, and fruits, respectively, which were kept in a vacuum desiccator until constant weight.

### 2.3. HPLC Analyses

In the HPLC analyses, an exploratory gradient elution was used [1, 9, 10]. Fingerprints were registered by RP-HPLC-DAD on a Waters 2695 apparatus equipped with a UV-DAD detector (Waters 2996). A LiChrospher 100 RP-18 column (5 *μ*m, 250 × 4 mm i.d.; Merck, Darmstadt, Germany) was employed at 40°C, flow rate of 1.0 mL/min, and detection at wavelengths of 220, 280, and 350 nm. To an aliquot 10.0 mg of dried extract/fractions and 1.0 mg of each of the isolated compounds HPLC grade methanol was added and the mixture was dissolved by sonication in an ultrasound bath for 15 min, followed by centrifugation at 10,000 rpm for 10 min. The supernatant was filtered through a Millipore membrane (0.2 *μ*m) and injected (10.0 *μ*L) onto the equipment. Elution was carried out with a linear gradient of water (a) and acetonitrile (b) (from 5% to 95% of B in 60 min).

### 2.4. Isolation of Chemical Components from Leaves Extract

To a portion of EEFFL (10.0 g), MeOH was added, and an insoluble precipitate was separated by filtration through sintered glass funnel and washed thoroughly with MeOH yielding 2.8 g. The precipitate was recrystallized out from methanol/water (1 : 1) giving 1.9 g of compound** 1**. The filtrate was dried in a rotary evaporator under reduced pressure at 50°C, leaving a dark residue (FFLMW, 7.0 g). AFLMW was subjected to fractionation over a silica gel column with n-hexane/CH_2_Cl_2_ (1 : 1), CH_2_Cl_2_, CH_2_Cl_2_/EtOAc (1 : 1), EtOAc/MeOH (2 : 1), EtOAc/MeOH (1 : 2), MeOH, MeOH/H_2_O (2 : 1), and MeOH/H_2_O (1 : 2) as eluents. A portion of EtOAc/MeOH (2 : 1) fraction 2 (1.0 g) was filtered over a Sephadex LH 20 column with MeOH as eluent, giving 12 fractions. Fractions 11 and 12 were combined leading to a yellow solid (554.0 mg) which was further purified by HPLC employing Shim-pack PRC-ODS column (20 mm i.d. × 25 cm, flow rate 5.0 ml/min) and a gradient elution with MeOH/H_2_O 45 to 95% for 50 minutes. Compounds** 2** (22.3 mg) and** 3** (56.1 mg) were isolated.

### 2.5. Isolation of Chemical Components from Stem Extract

To a portion of EEFFS (10.0 g), cold MeOH was added giving a precipitate which was separated by filtration on sintered glass funnel and washed thoroughly with MeOH yielding 2.43 g. The precipitate was recrystallized out twice from MeOH to give 405.0 mg of compound** 1**. The mother liquor from the second recrystallization was dried in a rotary evaporator under reduced pressure at 50°C, leaving a yellow residue (FFSMW2, 1.2 g) that was further subjected to fractionation by HPLC employing Shim-pack PRC-ODS column (20 mm i.d. × 25 cm, flow rate 5.0 ml/min) and a gradient elution with MeOH/H_2_O 45 to 95% for 50 minutes affording compound** 4** (511.0 mg).

### 2.6. Isolation of Chemical Components from Fruit Extract

To a portion of EEFFF (1.0 g), MeOH was added and an insoluble precipitate was separated by filtration on sintered glass funnel and washed thoroughly with methanol. The precipitate was subjected to recrystallization to give compound** 1 **(80.7 mg.) The filtrate was dried in a rotary evaporator under reduced pressure at 50°C, leaving a yellow residue (FFFMW, 809.7 mg) that was further purified by HPLC employing Shim-pack PRC-ODS column (20 mm i.d. × 25 cm, flow rate 5.0 ml/min) and a gradient elution with MeOH/H_2_O 45 to 95% for 50 minutes leading to the isolation of compounds** 1 **(96.3 mg),** 2 **(13.4 mg),** 3** (82.9 mg), and** 5** (84.6 mg).

### 2.7. Cell Culture and Virus

Vero cells (ATCC CCL-81) and LLCMK_2_ cells were cultured in Dulbecco's modified Eagle's medium (DMEM, Cultilab, Campinas, SP, Brazil) at 37°C, in 5% CO_2_ atmosphere, supplemented with 5% fetal bovine serum, 50 *μ*g/mL gentamicin, 100 U/mL penicillin, and 5 *μ*g/mL amphotericin B [1].

HSV-1 was obtained from the collection of Laboratório de Virus, UFMG, Belo Horizonte, Brazil. DENV-2, EMCV, and VACV-WR were kindly donated by Dr. L. Figueiredo (USP, Ribeirão Preto, Brazil), Dr. I. Kerr (London Research Institute, London, UK), and Dr. C. Jungwirth (University of Würzburg, Würzburg, Germany), respectively. The viruses were titrated by TCID_50_ in Vero cells [13] and the titers were 2.5 × 10^6^; 1.0 × 10^6^; 1.0 × 10^6^; and 1.0 × 10^4^ TCID_50_/mL, respectively, for HSV-1, EMCV, VACV-WR, and DENV-2.

### 2.8. Cytotoxicity Assay

Vero and LLCMK_2_ cells were exposed to different concentrations of extracts/fractions/compounds for 48 and 72 h [1]. After incubation, cell viability was assessed by the 3-(4,5-dimethylthiazol-2-yl)-2,5-diphenyltetrazolium bromide (MTT, Merck) assay at a concentration of 2 mg/mL in PBS [1, 14]. Each sample was assayed in four replicates for concentrations ranging from 500 to 0.125 *μ*g/mL. The cytotoxicity of each sample was expressed as CC_50_, that is, the concentration of sample that inhibited cell growth by 50% [1].

### 2.9. Antiviral Assays

The antiviral activity (EC_50_) of extracts∖fractions∖compounds was evaluated by the MTT assay [15]. Acyclovir (Calbiochem, Merck Brasil, São Paulo, SP, Brazil) and *α*-2a interferon (Bergamo Brasil, São Paulo, SP, Brazil) were used as positive controls [1]. The cell monolayer was infected with viral suspensions with titers of 2.5 × 10^6^; 1.0 × 10^6^; 1.0 × 10^6^; and 1.0 × 10^4^ TCID_50_/mL, respectively, for HSV-1, EMCV, VACV-WR, and DENV-2 [1]. Dilutions of the extracts, fractions, and compounds in noncytotoxic concentrations were added to the wells after viral infection. The plates were incubated at 37°C in humidified 5% CO_2_ atmosphere for a period of 48 and/or 72 h [1]. Experiments were carried out with eight different concentrations within the inhibitory range of the samples. The 50% inhibitor concentrations of the viral effect (EC_50_) for each of the extracts, fractions, and constituents were calculated from concentration-effect-curves after no linear regression analysis [1]. The selectivity index (SI) is defined as CC_50_ over EC_50_. Statistical calculations were carried out with the GraphPad prism 5.0 software package (Statistica). Results are expressed as the mean ± SEM of 4 independent experiments. Student's* t*-test was used for statistical analyses; *P* values > 0.05 were considered to be significant.

### 2.10. Structural Determination

The compounds isolated were identified on the basis of spectral analyses and comparison with literature data. ^1^D and ^2^D ^1^H and ^13^C-NMR spectra such as COSY, HSQC, and HMBC were obtained on a Bruker Avance DRX400 instrument in DMSO-d6 with TMS as internal standard. Chemical shifts are given as *δ* (ppm). LC-MS data were obtained by electrospray ionization mass spectrometry (ESI-MS) in an Esquire 3000 Plus Bruker Daltonics equipment, capillary: 4000 V, nebulizer: 27 psi, dry gas: 7.0 L/min, dry temp: 320°C, and mass flux 100 * *uL/min, in the Central Analítica, Instituto de Química, Universidade de São Paulo, São Paulo, SP, Brazil [1].

### 2.11. Spectroscopic Data for Isolated Compounds

*Mangiferin (* ***1*** *; 2-β-D-Glucopyranosyl-1,3,6,7-tetrahydroxy-9H-xanthen-9-one)*. orange powder (MeOH); m.p. decomposes at 265.0–275.0; Lit. 271–274°C [16]; UV (MeOH) *λ*_max_ 239, 258, 316, 365 nm; IR *ν*_max_ 3363, 3184, 2935, 2891, 1648, 1619, 1592, 1565, 1520, 1490, 1462, 1406, 1351, 1294, 1251, 1191, 1093, 1074, 1032, 878, 822, 735 cm^−1^; ^1^H NMR (DMSO-d6, 400 MHz): *δ* 13.77 (s, 1H, 1-OH), 10.55 (s, 2H, 6,7-OH), 7.38 (s, 1H, H-8), 6.86 (s, 1H, H-5), 6.37 (s, 1H, H-4), 4.87 (s, 2H, 3′,4′-OH), 4.61 (d, 10.0 Hz, 1H, H-1′), 4.49 (s, 1H, 6′-OH), 4.05 (t, 8.4 Hz, 1H, H-2′), 3.70 (d, 11.2 Hz, 1H, H-6′_B_), 3.41 (m, 1H, H-6′_A_), 3.18 (m, 1H, H-3′), 3.18 (m, 1H, H-4′), 3.18 (m, 1H, H-5′). ^13^C NMR (DMSO-d6, 100 MHz): *δ* 179.1 (C, C-9), 163.8 (C, C-3), 161.8 (C, C-1), 156.2 (C, C-4a), 154.0 (C, C-6), 150.8 (C, C-10a), 143.7 (C, C-7), 111.7 (C, C-8a), 108.1 (C, C-8), 107.5 (C, C-2), 102.6 (C, C-5), 93.3 (C, C-4), 81.5 (C, C-5′), 79.3 (C, C-3′), 73.1 (C, C-1′), 70.6 (C, C-4′), 70.3 (C, C-2′), 61.5 (C, C-6′); HRESI-MS* m/z* 423.1024 [M − H]^−^ (calcd for C_19_H_19_O_11_, 423.0927).

*2*′*-O-trans-caffeoylmangiferin ( ****2****; 2-(2*′*-O-trans-caffeoyl)-C-β-D-glucopyranosyl-1,3,6,7-tetrahydroxyxanthone)*. orange powder (MeOH); m.p. decomposes at 269.0–278.0°C; UV (MeOH) *λ*_max_ 232, 258, 315, 365 nm; IR *ν*_max_ 3217, 1690, 1604, 1514, 1471, 1259, 1150, 1071, 812 cm^−1^; ^1^H NMR (DMSO-d6, 400 MHz): *δ* 13.83 (s, 1H, 1-OH), 7.33 (s, 1H, H-8), 7.23 (d, 16.0 Hz, 1H, H-7′′), 6.92 (m, 1H, H-2′′), 6.87 (d, 8.0 Hz, 1H, H-6′′), 6.80 (s, 1H, H-5), 6.71 (d, 8.0 Hz, 1H, H-5′′), 6.30 (s, 1H, H-4), 6.01 (d, 16.0 Hz, 1H, H-8′′), 5.65 (m, 1H, H-2′), 4.95 (d, 12.0 Hz, 1H, H-1′), 3.76 (m, 1H, H-6′_B_), 3.74 (m, 1H, H-6′_A_), 3.49 (m, 1H, H-3′), 3.45 (m, 1H, H-4′), 3.27 (m, 1H, H-5′). ^13^C NMR (DMSO-d6, 100 MHz): *δ* 179.0 (C, C-9), 165.2 (C, C-9′′), 164.4 (C, C-3), 161.5 (C, C-1), 156.3 (C, C-4a), 154.5 (C, C-6), 150.8 (C, C-10a), 148.2 (C, C-4′′), 145.9 (C, C-3′′), 144.9 (C, C-7′′), 143.9 (C, C-7), 125.4 (C, C-1′′), 121.6 (C, C-6′′), 115.7 (C, C-5′′), 115.0 (C, C-2′′), 114.2 (C, C-8′′), 111.4 (C, C-8a), 107.8 (C, C-8), 105.7 (C, C-2), 102.5 (C, C-5), 94.2 (C, C-4), 82.3 (C, C-5′), 76.9 (C, C-3′), 72.4 (C, C-2′), 71.1 (C, C-1′), 71.1 (C, C-4′), 61.9 (C, C-6′); HRESI-MS* m/z* 583.1083 [M − H]^−^ (calcd for C_28_H_23_O_14_, 583.1088).

*2*′*-O-trans-coumaroylmangiferin ( ****3****; 2-(2*′*-O-trans-coumaroyl)-C-β-D-glucopyranosyl-1,3,6,7-tetrahydroxyxanthone)*. orange powder (MeOH); m.p. decomposes at 271.0–285.0; UV (MeOH) *λ*_max_ 233, 258, 315, 364 nm; IR *ν*_max_ 3255, 1694, 1614, 1472, 1417, 1365, 1284, 1230, 1150, 1079, 1030, 996, 815, 765, 706, 681 cm^−1^; ^1^H NMR (DMSO-d6, 400 MHz): *δ* 13.86 (s, 1H, 1-OH), 7.39 (s, 1H, H-8), 7.37 (d, 16.0 Hz, 1H, H-7′′), 7.35 (d, 8.0 Hz, 1H, H-2′′), 7.35 (d, 8.0 Hz, 1H, H-6′′), 6.81 (s, 1H, H-5), 6.76 (d, 8.0 Hz, 1H, H-3′′), 6.76 (d, 8.0 Hz, 1H, H-5′′), 6.32 (s, 1H, H-4), 6.10 (d, 16.0 Hz, 1H, H-8′′), 5.56 (m, 1H, H-2′), 5.01 (d, 8.0 Hz, 1H, H-1′), 3.78 (d, 8.0 Hz, 1H, H-6′_B_), 3.66 (m, 1H, H-6′_A_), 3.64 (m, 1H, H-3′), 3.47 (t, 10.0 Hz, 1H, H-4′), 3.42 (m, 1H, H-5′). ^13^C NMR (DMSO-d6, 100 MHz): *δ*, 165.8 (C, C-9′′), 164.0 (C, C-3), 160.0 (C, C-1), 160.0 (C, C-4′′), 158.7 (C, C-4a), 154.0 (C, C-6), 151.3 (C, C-10a), 144.5 (C, C-7′′), 143.9 (C, C-7), 130.1 (C, C-2′′), 130.1 (C, C-6′′), 125.4 (C, C-1′′), 115.9 (C, C-3′′), 115.9 (C, C-5′′), 114.4 (C, C-8′′), 112.2 (C, C-8a), 108.3 (C, C-8), 105.5 (C, C-2), 102.8 (C, C-5), 94.0 (C, C-4), 81.4 (C, C-5′), 76.8 (C, C-3′), 72.5 (C, C-2′), 71.4 (C, C-1′), 70.7 (C, C-4′), 61.4 (C, C-6′); HRESI-MS* m/z* 567.1144 [M − H]^−^ (calcd for C_28_H_23_O_13_, 567.1139).

*Chrysin (* ***4*** *; 5,7-Dihydroxy-2-phenyl-4H-1-benzopyran-4-one)*. orange powder (MeOH); m.p. 285.6–287.9°C; Lit. 289–291°C [17]; UV (MeOH) *λ*_max_ 267, 313 (sh) nm; IR *ν*_max_ 3283, 2920, 1690, 1610, 1512, 1444, 1341, 1230, 1171, 1066, 1037, 1012, 893, 823 cm^−1^; ^1^H NMR (DMSO-d6, 400 MHz): *δ* 12.0 (s, 1H, 5-OH); 8.06 (dd, 6.4 and 1.6 Hz, 2H, H-2′ and H-6′), 7.55–7.64 (m, 3H, H-3′, H-4′ and H-5′); 6.95 (s, 1H, H-3); 6.86 (s, 1H, 7-OH); 6.53 (d, 2 Hz, 1H, H-8); 6.23 (d, 2 Hz, 1H, H-6). ^13^C NMR (DMSO-d6, 100 MHz): *δ* 181.9 (C, C-4), 164.4 (C, C-7), 163.2 (C, C-2), 161.5 (C, C-5), 157.5 (C, C-9), 132.0 (C, C-4′), 130.7 (C, C-1′), 129.4 (C, C-5′), 129.1 (C, C-3′), 126.4 (C, C-2′), 126.4 (C, C-6′), 105.2 (C, C-3), 104.0 (C, C-10), 99.0 (C, C-6), 94.1 (C, C-8); HRESI-MS* m/z* 255.0671 [M + H]^+^ (calcd for C_15_H_11_O_4_, 255.0657).

*2*′*-O-trans-cinnamoylmangiferin ( ****5****; 2-(2*′*-O-trans-cinnamoyl)-C-β-D-glucopyranosyl-1,3,6,7-tetrahydroxyxanthone)*. orange powder (MeOH); m.p. decomposes at 269.0–279.0 UV (MeOH) *λ*_max_ 222, 257, 275 (sh), 320 (sh), 366 nm; IR *ν*_max_ 3252, 1693, 1614, 1471, 1417, 1183, 1150, 1079, 815, 765, 706 cm^−1^; ^1^H NMR (DMSO-d6, 400 MHz): *δ* 13.87 (s, 1H, 1-OH), 7.51 (m, 1H, H-3′′), 7.51 (m, 1H, H-5′′), 7.33 (s, 1H, H-8), 7.23 (d, 16.0 Hz, 1H, H-7′′), 6.80 (s, 1H, H-5), 6.36 (m, 1H, H-2′′), 6.36 (m, 1H, H-6′′), 6.32 (s, 1H, H-4), 6.31 (d, 16.0 Hz, 1H, H-8′′), 5.55 (m, 1H, H-2′), 5.04 (d, 12.0 Hz, 1H, H-1′), 3.78 (dd, 12.0, 2.5 Hz, 1H, H-6′_B_), 3.66 (dd, 12.0, 4.0 Hz, 1H, H-6′_A_), 3.64 (m, 1H, H-3′), 3.51 (m, 1H, H-4′), 3.44 (m, 1H, H-5′). ^13^C NMR (DMSO-d6, 100 MHz): *δ* 179.3 (C, C-9), 165.0 (C, C-9′′), 163.4 (C, C-3), 161.0 (C, C-1), 156.8 (C, C-4a), 153.9 (C, C-6), 151.1 (C, C-10a), 143.9 (C, C-7′′), 143.4 (C, C-7), 133.9 (C, C-1′′), 129.9 (C, C-4′′), 128.5 (C, C-2′′), 128.5 (C, C-6′′), 127.7 (C, C-3′′), 127.7 (C, C-5′′), 117.6 (C, C-8′′), 111.9 (C, C-8a), 107.8 (C, C-8), 104.9 (C, C-2), 102.3 (C, C-5), 101.3 (C, C-9a), 94.0 (C, C-4), 80.9 (C, C-5′), 75.9 (C, C-3′), 72.4 (C, C-2′), 71.1 (C, C-1′), 70.2 (C, C-4′), 60.9 (C, C-6′); HRESI-MS* m/z* 551.1152 [M − H]^−^ (calcd for C_28_H_23_O_12_, 551.1090).

## 3. Results

### 3.1. HPLC Analyses, Isolation, and Identification of Compounds from* Fridericia formosa*

**Figure 1:**
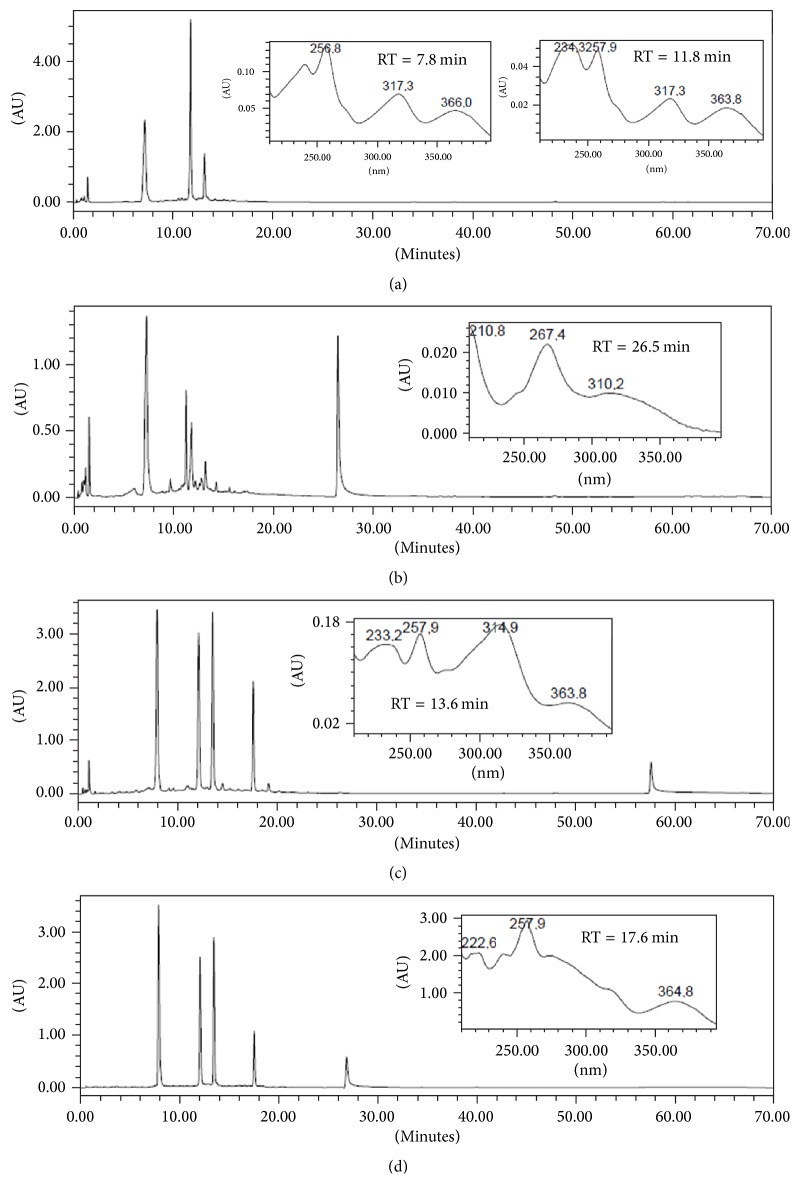
RP-HPLC-DAD fingerprints for the crude ethanol extracts from (a) ethanol extract from* Fridericia formosa* leaves (EEFFL) with mangiferin (RT = 7.8 min) and 2′-*O*-*trans*-caffeoylmangiferin (RT = 11.8 min) UV spectrum registered online detection 350 nm, (b) ethanol extract from* Fridericia formosa* stems (EEFFS) with UV spectra registered online for peak corresponding to chrysin (RT = 26.8 min), (c) ethanol extract from* Fridericia formosa* fruits (EEFFF) with UV spectra registered online for peak corresponding to 2′-*O-trans-*coumaroylmangiferin (RT = 13.6 min), and (d) for a mixture of the isolated compounds with UV spectra registered online for peak corresponding to 2′-*O*-*trans-*cinnamoylmangiferin (RT = 17.6 min). Detection: 350 nm. Chromatographic conditions: see Experimental.

HPLC-DAD analyses allowed identifying xanthones as major constituents in all the extracts, as inferred from their UV spectra that were registered online (Figure 1). UV spectra of 1,3,6,7-tetraoxygenated xanthones are characterized by the presence of three or more absorption bands of decreasing intensity [18]. A compound with retention time (RT) of 7.8 min was detected in all the ethanol extracts as the major constituent in extracts of stems and fruits.

**Figure 2:**
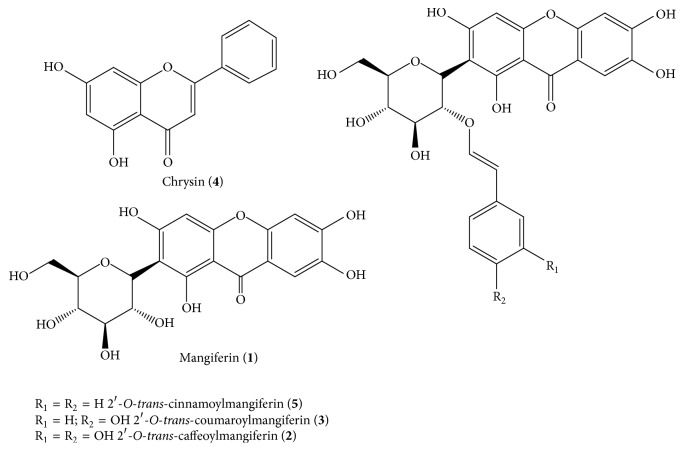
Chemical structures of mangiferin (**1**), 2′-*O*-*trans*-caffeoylmangiferin (**2**), 2′-*O-trans-*coumaroylmangiferin (**3**), chrysin (**4**), and 2′-*O*-*trans-*cinnamoylmangiferin (**5**).

Bioguided fractionation of* F. formosa *ethanol extracts led to the isolation of five compounds (Figure 2) which were identified by comparison with literature spectroscopic data (^1^H and ^13^C NMR, DEPT-135 experiment, COSY, HMQC, HMBC, IR, and MS). From the EtOH extract of leaves three compounds (1–3) were obtained. Compound** 1** was identified as mangiferin, a C-glucosylxanthone (*λ*_max_ 239, 258, 316, and 365 nm, HPLC-DAD online). Its identification was confirmed by HRMS and comparison of ^1^H and ^13^C NMR spectra with literature data [19]. Additionally, mangiferin (**1**) was also isolated from stems (EEAFS) and fruit (EEAFF) extracts. Compounds** 2** and** 3** were purified from leaves and fruits by Sephadex LH-20 gel filtration and preparative HPLC. The presence of the mangiferin chromophore for both compounds was indicated by their UV spectra. The protonated molecular ions [M + H]^+^ for** 2** and** 3** (*m/z* 585.1084 and* m/z* 569.1144, resp.) were determined by accurate positive HRESI-MS. A comparative analysis of fragment ions detected in experiments by HPLC/ESI-MS∖MS showed that the only noticeable differences were associated with the cinnamic acid moieties attached to the mangiferin unity, caffeic acid in** 2, **and* p*-coumaric acid in** 3**. The isolation of mangiferin cinnamic esters from* Fridericia samydoides* and* F. patellifera* was previously reported [20, 21] and a comparative analysis of these NMR data confirmed the identification of compounds** 2** and** 3** as 2′-*O-trans-*caffeoylmangiferin and 2′-*O-trans*-coumaroylmangiferin, respectively. The position of the cinnamic ester group in the glucose unity was proved by long-range HMBC correlation between the ester carbonyl group and the glucosyl hydrogen: *δ*_C_ 165.2 and H-2′*δ*_H_ 5.65, in compound** 2**, and *δ*_C_ 165.8 and H-2′  *δ*_H_ 5.56, in compound** 3**.

Additional quantity of mangiferin (**1**) was obtained as an unsoluble fraction when methanol was added to the dried stem ethanol extract. Fractionation of the filtrate (AFSMW2) by preparative RP-HPLC afforded compound** 4** whose spectral data (UV, IR, ^1^H and ^13^C NMR) and comparison with literature data [22] allowed its identification as the flavonoid chrysin (**4**).

Finally, column chromatographic fractionation of the ethanol fruit extract afforded four C-glucosylxanthones which were shown to be identical to those obtained from the leaves extract, mangiferin (**1**), caffeoyl mangiferin (**2**), and coumaroyl mangiferin (**3**), besides compound** 5**, and MM 542 Da, which might correspond to a mangiferin cinnamic ester, was confirmed by NMR data. Two regioisomers, 2′-*O-trans*-cinnamoylmangiferin and 3′-*O-trans*-cinnamoylmangiferin, have been previously isolated from stems and leaves extracts of* F. samydoides* and* F. patellifera*, respectively [20, 21]. A comparison of ^13^C and ^1^H NMR data, including two-dimensional COSY, HSQC, and HMBC, with those previously reported [20] allowed the identification of compound** 5 **as 2′-*O-trans*-cinnamoylmangiferin.

### 3.2. Bioguided Fractionation of Leaves, Stems, and Fruits Ethanol Extracts from* Fridericia formosa*

**Figure 3:**
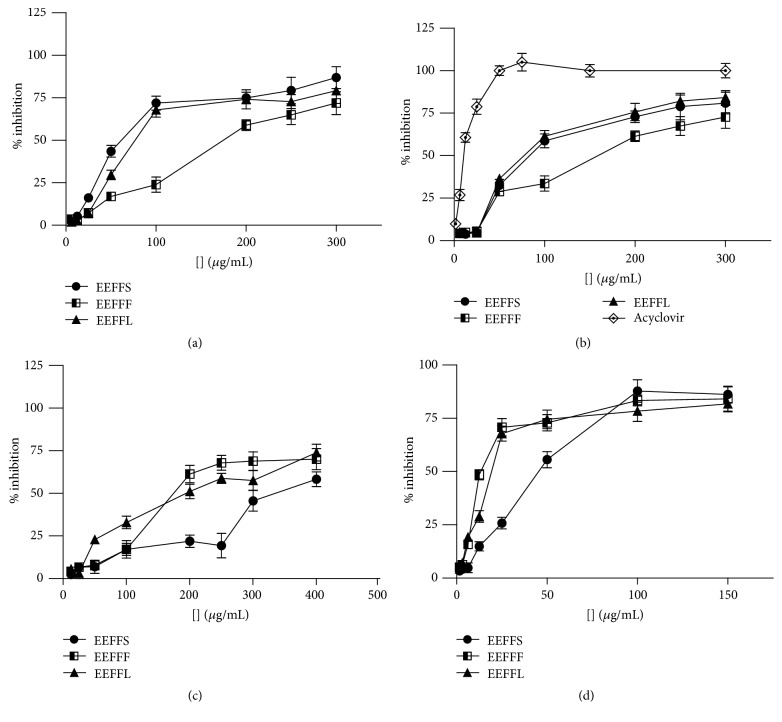
Dose-response curves for antiviral activity of ethanol extracts from* Fridericia formosa* (a) leaves (EEFFL), stems (EEFFS), and fruits (EEFFF) against VAC-WR; (b) EEFFL, EEFFS, and EEFFF against HSV-1; (c) EEFFL, EEFFS, and EEFFF against EMCV; (d) EEFFL, EEAFS, and EEFFF against DENV-2.

Confirming previously published results [8], the ethanol extracts from leaves (EEFFL), stems (EEFFS), and fruits (EEFFF) of* F. formosa* showed antiviral activity against EMCV, HSV-1, and VACV-WR with EC_50_ values in the range of 85.6 ± 4.1 to 147.8 ± 2.4 *μ*g/ mL (Table 1 and Figure 3). Furthermore, these extracts were evaluated against DENV-2 and disclosed good antidengue activity with EC_50_ values ranging from 13.1 ± 1.6 to 42.6 ± 2.3 *μ*g/mL (Table 1).

**Figure 4:**
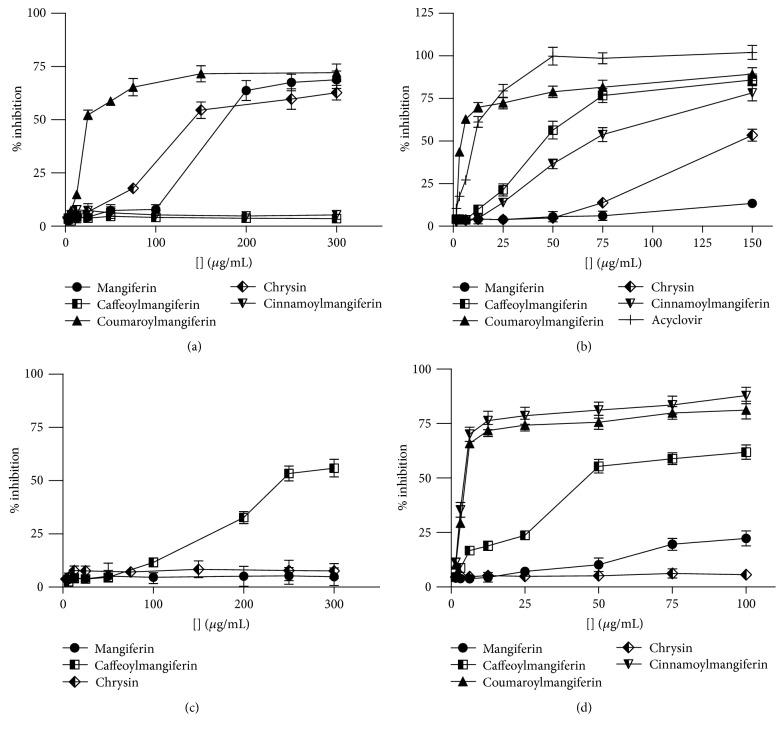
Dose-response curves for antiviral activity of mangiferin (**1**), 2′-*O*-*trans*-caffeoylmangiferin (**2**), 2′-*O-trans-*coumaroylmangiferin (**3**), chrysin (**4**), and 2′-*O*-*trans-*cinnamoylmangiferin (**5**). (a) Against VAC-WR; (b) against HSV-1; (c) against EMCV; (d) against DENV-2.

An aliquot of the leaves extract (EEFFL, 10.0 g) was submitted to bioguided fractionation. Initially, addition of cold methanol to EEFFL led to mangiferin (**1**), as an unsoluble fraction (1.9 g), and to a methanol soluble fraction (FFLMW). Mangiferin (**1**) was tested against all the four virus samples and showed a low antiviral effect (Table 1). FFLMW was subjected to a chromatographic fractionation through a silica gel column employing as eluents n-hexane/CH_2_Cl_2_ (1 : 1), CH_2_Cl_2_, CH_2_Cl_2_/EtOAc (1 : 1), EtOAc/MeOH (2 : 1), EtOAc/MeOH (1 : 2), MeOH, MeOH/H_2_O (2 : 1), and MeOH/H_2_O (1 : 2) fractions that were assayed against DENV-2, EMCV, HSV-1, and VACV-WR. Three fractions were active against HSV-1. The EtOAc/MeOH (2 : 1) fraction was the only one active against VACV-WR and EMCV. Best results were observed against DENV-2 for four active fractions with EC_50_ ranging from 3.9 ± 0.4 to 41.8 ± 5.6 *μ*g/mL (Table 1). Fractionation of the EtOAc/MeOH (2 : 1) fraction through a Sephadex LH20 column and preparative RP-HPLC afforded two xanthones: 2′-*O-trans*-caffeoylmangiferin (**2**) and 2′-*O-trans-*coumaroylmangiferin (**3**). Xanthone** 2 **showed high activity against DENV-2, HSV-1, and VACV-WR (EC_50_ of 4.1 ± 0.4 *μ*g/mL, 4.6 ± 1.5 *μ*g/mL, and 23.8 ± 1.0 *μ*g/mL, resp.) while 2′-*O-trans-*coumaroylmangiferin (**3**) was active against DENV-2, EMCV, and HSV-1 but with lower EC_50_ values (Table 1 and Figure 4).

EEFFS (10.0 g) was also submitted to bioguided fractionation. Initially, addition of cold methanol to the crude ethanol extract led to the separation of mangiferin (**1**) (405.0 mg), as the unsoluble fraction. Fractionation of the methanol soluble fraction (FFCMW) by preparative RP-HPLC afforded chrysin (**4**) that showed low activity against HSV-1 and VACV-WR (Table 1 and Figure 4).

Finally, bioguided fractionation of EEFFS (10.0 g) by extraction with cold methanol led to mangiferin (**1**) (80.7 mg), as an unsoluble part, and FFFMW (methanol soluble fraction) that, on fractionation by preparative RP-HPLC, afforded mangiferin (**1**), 2′-*O-trans*-caffeoylmangiferinn (**2**), 2′-*O-trans-*coumaroylmangiferin (**3**), and 2′-*O*-*trans-*cinnamoylmangiferin (**5**). The last compound (**5**) was highly active against DENV-2 virus (EC_50_ 3.5 ± 0.5 *μ*g/mL) and moderately active against HSV-1 (EC_50_ 77.4 ± 4.3) (Table 1 and Figure 4).

## 4. Discussion

Phytochemical investigation of EtOH extracts of leaves, stems, and fruits from* F. formosa* led to the isolation of five compounds. Four of them were identified as C-glucosylxanthones, namely, mangiferin (**1**), along with three cinnamoyl esters of mangiferin (**2**,** 3**,** and 5**), and one flavonoid, chrysin (**4**) (Figure 2). Chrysin (**4**) was isolated from the stems extract and showed low antiviral activity against VACV-WR and HSV-1 with EC_50_ > 100 *µ*g/ml and did not inhibit the replication cycle of DENV-2 and EMCV. Good activity of this flavonoid against HSV-1 with an EC_50_ 2.5 *µ*M was previously reported [23] and our negative result might be related to difference in susceptibility of strains. This flavonoid is present in other species of the Bignoniaceae family, such as* F. samydoides *[24]. Previously isolated from* Oroxylum indicum* (Bignoniaceae), it is reported as disclosing in vitro anti-inflammatory and anticancer effects [25–27]. Marketed as a bodybuilding supplement it is claimed to increase testosterone levels or stimulate testosterone production; however, clinical tests have shown no effect on testosterone levels in men [28].

As shown in Figure 2, the four xanthones isolated from the extracts of leaves, stems, and fruits of* A. formosa *were identified as mangiferin (**1**), 2′-*O-trans-*caffeoylmangiferin (**2**), 2′-*O-trans-*coumaroylmangiferin (**3**), and 2′-*O-trans*-cinnamoylmangiferin (**5**). The antiviral effect of these compounds was evaluated against DENV-2, EMCV, HSV-1, and VACV-WR (Figure 3). Excepting mangiferin (**1**), the other three xanthones showed good antiviral effects and inhibited the replication cycle of DENV-2, HSV-1, and VACV-WR (Table 1 and Figure 4).

Mangiferin (**1**) is mainly obtained from mango tree* (Mangifera indica)* and preclinical studies showed that it exhibits antidiabetic, antioxidant, antiviral, cardiotonic, hypotensive, and anti-inflammatory properties [29]. The biological activities of mangiferin have been attributed to modulating expression of a large number of genes that are critical for the regulation of apoptosis, viral replication, inflammation, and various autoimmune diseases [30]. Furthermore mangiferin disclosed low cytotoxicity and good inhibitory activity on HIV-1 replication in a dose dependent manner [30, 31]. Mechanism studies revealed that mangiferin might inhibit the HIV-1 protease and is, therefore, a novel nonpeptide protease inhibitor of HIV protease [30, 31].

Recent data on biological activity of mangiferin cinnamoyl esters showed that they are antioxidant agents similar to mangiferin and are antiplasmodial with moderate activity in vitro against* Plasmodium falciparum* 3D7 clone, which is chloroquine-sensitive (IC_50_ 18.1 to 26.5 *µ*M) [21].

Recently, a total of twenty xanthones were isolated from* Swertia mussotii* (Gentianaceae) and their antiviral activity was evaluated [32]. Eight of these xanthones exhibited significant activity against hepatitis B virus inhibiting DNA replication with EC_50_ values from 0.01 mM to 0.13 mM [32]. Additionally, the xanthones norbellidifolin, 1,5,8-trihydroxy-3-methoxyxanthone, and 2-*C-β-D*-glucopyranosyl-1,3,7-trihydroxyxanthone showed remarkable activity with EC_50_ values of 0.77, > 0.98, and 0.21 mM for hepatitis B surface antigen (HBsAg) and <0.62, 0.35, and 0.04 mM for hepatitis B antigen (HBeAg), respectively [32]. Besides, euxanthone, from* Garcinia oblongifolia* (Clusiaceae) leaves extract, disclosed significant activity in vitro against* Enterovirus* 71, with EC_50_ value of 12.2 *μ*M [33]. In addition, the selectivity index of this compound was 3.0 in relation to the cytotoxicity to Vero cells (CC_50_ 36.6 *μ*M) [33]. Molecular docking studies of 272 xanthones for interactions with a group of seven fungal and two viral enzymes showed that prenylated xanthones are important hits for inhibition of the selected enzymes [34]. In general, prenylated xanthones were able to establish significantly stronger complexes with the tested enzymes [34]. Some compounds were pointed out as potential inhibitors for those enzymes, including nigrolineaxanthone and latisxanthone D as probably potent inhibitors of HIV-1 reverse transcriptase [34]. Xanthones have important advantages as potential antiviral agents because of their availability as natural compounds and the possibility of being easily synthesized and also for the demonstrated interaction with some important microorganisms targets [34].

## 5. Conclusions

Our results reveal that* F. formosa* is a rich source of mangiferin (**1**) (≅47 g·kg^−1^ of dry leaves), a C-glucosyl xanthone with several therapeutic and cosmetic uses [30]. Its content in* F. formosa* is higher than in mango tree* (Mangifera indica)*, its usual source (≅1.7 g·kg^−1^ of dry peel) [35]. However, mangiferin was practically inactive against the virus assayed. On the other hand, minor constituents, represented by mangiferin cinnamoyl esters, seem to be the main responsible constituents for the antiviral activity previously reported for extracts of different botanical parts of this species [8]. Special attention is called for the IS of these xanthone derivatives, particularly for the caffeoyl (**2**) and cinnamoyl (**5**) esters, with IS > 100. Our findings are the first report on the chemical and antiviral activity of* F. formosa *constituents. Our results are in line with the traditional use of* Fridericia* species as anti-infectious agents in different South American countries [1, 9] and might be of interest for the development of standardized antiviral phytomedicines.

## Figures and Tables

**Table 1:** Cytotoxicity (CC_50_, Vero, and LLCMK_2_ cells), in vitro antiviral activity (EC_50_), and selectivity index (SI) for ethanol extracts from *Fridericia formosa* leaves (EEFFL), stems (EEFFS), fruit (EEFFF), fractions, and compounds 1–5.

Extracts/fractions/compound	Vero CC_50_ *μ*g/mL	LLCMK_2_ CC_50_ *μ*g/mL	^a^HSV-1 EC_50_ *μ*g/mL	SI	^b^VACV-WR EC_50_ *μ*g/mL	SI	^c^EMCV EC_50_ *μ*g/mL	SI	^d^DENV-2 EC_50_ *μ*g/mL	SI
EEFFS	>500	173.9 ± 9.8	93.2 ± 5.4	>5.4	59.2 ± 2.4	>8.4	322.5 ± 14.4	>1.5	42.6 ± 2.3	4.1
EEFFF	>500	>500	147.8 ± 2.4	>3.4	252.7 ± 3.9	>2.0	134.4 ± 5.9	>3.7	13.1 ± 1.6	>38.2
EEAFL	>500	>500	85.6 ± 4.1	>5.8	83.7 ± 3.1	>6.0	199.4 ± 13.8	>2.5	16.3 ± 6.8	>30.7
FFHDF (1 : 1)	222.0 ± 7.3	86.7 ± 8.5	NA		NA		NA		NA	
FFDF	263.3 ± 13.9	95.1 ± 9.3	NA		NA		NA		NA	
FFDEF (1 : 1)	50.7 ± 2.5	13.8 ± 2.1	NA		NA		NA		3.9 ± 0.36	3.5
FFEF	>500	>500	NA		NA		NA		NA	
FFEMF (1 : 1)	>500	>500	169.7 ± 21.0	>2.9	182.9 ± 11.4	>2.7	190.5 ± 14.7	>2.6	31.8 ± 5.7	>15.7
FFMF	>500	>500	50.3 ± 2.8	>9.9	NA		NA		41.8 ± 5.6	>12.0
FFMWF (2 : 1)	>500	>500	35.7 ± 2.0	>14.0	NA		NA		22.8 ± 0.8	>21.9
FFMWF (1 : 2)	>500	>500	NA		NA		NA		NA	
Mangiferin (**1**)	>500	>500	267.9 ± 6.7 (634.8 ± 15.9)	>1.9	182.7 ± 14.3 (432.5 ± 33.9)	>2.7	NA		265.5 ± 14.0 (629.1 ± 33.2)	>1.9
2′-*O-Trans*-caffeoylmangiferin (**2**)	>500	>500	4.6 ± 1.5 (7.9 ± 2.6)	>108.7	23.8 ± 1.0 (40.7 ± 1.7)	>21.0	NT		4.1 ± 0.4 (7.0 ± 0.7)	>121.9
2′-*O-Trans*-coumaroylmangiferin (**3**)	>500	>500	47.4 ± 6.1 (83.4 ± 10.7)	>10.5	NA		241.0 ± 31.8 (424.3 ± 56.0)	>2.1	40.4 ± 4.2 (71.1 ± 7.4)	>12.4
Chrysin (**4**)	>500	>500	146.3 ± 15.9 (575.9 ± 62.6)^e^	>3.4	123.5 ± 10.5 (486.2 ± 41.3)	>4.0	NA		NA	
2′-*O-Trans*-cinnamoylmangiferin (**5**)	>500	>500	77.4 ± 4.3 (140.2 ± 7.8)	>6.5	NA		NT		3.5 ± 0.5 (6.3 ± 0.9)	>148.9
*Acyclovir*	>1000	>1000	40^f^							
*Interferon α*	>^g^1.0 × 10^5^	>^g^1.0 × 10^5^			^fg^1.5 × 10^2^		^fg^2.5 × 10^3^		^fg^2.5 × 10^3^	

SI, selectivity index;  ^a^viral titer TCID_50_/mL 2.5 × 10^6^ in 48 h;  ^b^viral titer TCID_50_/mL 1.0 × 10^6^ in 48 h;  ^c^viral titer TCID_50_/mL 1.0 × 10^6^ in 48 h;  ^d^viral titer TCID_50_/mL 1.0 × 10^4^ in 72 h; NA, no activity in the assayed concentrations; NT, no test;  ^e^concentration in *µ*M;  ^f^80 to 100% inhibition of cytopathic effect;  ^g^concentration in UI/mL; EEFFL, ethanol extract from *Fridericia formosa* leaves; FFHDF, *Fridericia formosa* n-hexane/dichloromethane 1 : 1 fraction; FFDF, *Fridericia formosa* dichloromethane fraction; FFDEF, *Fridericia formosa* dichloromethane/ethyl acetate 1 : 1 fraction; FFEF, *Fridericia formosa* ethyl acetate fraction; FFEMF, *Fridericia formosa* ethyl acetate/methanol 1 : 1 fraction; FFMF, *Fridericia formosa* ethyl methanol fraction; FFMWF, *Fridericia formosa* methanol/water 2 : 1 fraction; FFMWF, *Fridericia formosa* methanol/water 1 : 2 fraction; *Fridericia formosa* fractions from chromatography of EEFFL over silica gel column.
